# Sustained humoral immunity in the patients recovered from severe fever with thrombocytopenia syndrome

**DOI:** 10.1186/s41182-025-00807-4

**Published:** 2025-09-30

**Authors:** Ryotaro Kubo, Rokusuke Yoshikawa, Yuji Fujii, Takumi Kawasaki, Takahiro Takazono, Koichi Izumikawa, Koya Ariyoshi, Hiroshi Mukae, Jiro Yasuda

**Affiliations:** 1 Department of Emerging Infectious Diseases, National Research Center for the Control and Prevention of Infectious Diseases (CCPID), Nagasaki University, Nagasaki, Japan; 2 Department of Emerging Viral Diseases, Graduate School of Biomedical Sciences, Nagasaki University, Nagasaki, Japan; 3 Department of Respiratory Medicine, Nagasaki University Graduate School of Biomedical Sciences, Nagasaki, Japan; 4 Department of Emerging Infectious Diseases, Institute of Tropical Medicine, Nagasaki University, Nagasaki, Japan; 5 Experimental Animal Research Division, National Research Center for the Control and Prevention of Infectious Diseases (CCPID), Nagasaki University, Nagasaki, Japan; 6 Department of Immune Dynamics in Viral Infections, National Research Center for the Control and Prevention of Infectious Diseases (CCPID), Nagasaki University, Nagasaki, Japan; 7 Department of Infectious Diseases, Nagasaki University Graduate School of Biomedical Sciences, Nagasaki, Japan; 8 Department of Clinical Medicine, Institute of Tropical Medicine, Nagasaki University, Nagasaki, Japan

**Keywords:** SFTS, Humoral immunity, Neutralizing antibody, Memory B cells

## Abstract

**Background:**

Severe fever with thrombocytopenia syndrome (SFTS) is a tick-borne viral disease with a mortality rate of 10–30%; however, effective vaccines and therapies for this disease have not yet been developed. Understanding the long-term immune response of recovered individuals is critical for vaccine development and treatment. In this study, we conducted an epidemiological investigation of antibody and memory B cell trends in individuals with SFTS.

**Methods:**

Peripheral blood mononuclear cells (PBMCs) and plasma were collected from 16 survivors of SFTS and five healthy controls. SFTS virus (SFTSV)-specific humoral immune responses were assessed using enzyme-linked immunosorbent assay (ELISA), biolayer interferometry (BLI), neutralization assays, and flow cytometry.

**Results:**

SFTSV Gn-specific IgG was detected in plasma samples from all patients using ELISA and BLI. All patient plasma samples also presented neutralizing activity against SFTSV infection, and the IC₅₀ values were correlated with ELISA OD values (*ρ* = 0.700, *P* = 0.003 and BLI signals (*ρ* = 0.818, *P* = 0.0002). Neutralizing antibodies and SFTSV Gn-specific memory B cells were detected in samples from patients up to 6.7 years post-infection.

**Conclusion:**

SFTSV-specific humoral immunity, including neutralizing antibodies and memory B cells, can persist in the majority of recovered patients, including those as late as 6.7 years post-infection. This information will be useful for the development of vaccines and antiviral therapies using antibodies against SFTS.

**Supplementary Information:**

The online version contains supplementary material available at 10.1186/s41182-025-00807-4.

## Background

SFTS is a tick-borne viral disease, which is caused by SFTS virus (SFTSV) (species name: *Bandavirus dabieense*) belonging to the genus *Banyangvirus* of the family *Phenuiviridae*. The associated clinical symptoms include thrombocytopenia, hepatic dysfunction, fever, and abnormal blood coagulation, and the fatality rate has been reported to range from 10–30% [1–3]. SFTSV is a negative-stranded RNA virus with three gene segments: large (L), medium (M), and small (S). The S, M, and L segments primarily encode nucleocapsid protein, membrane precursor protein (GP), and RNA-dependent RNA polymerase, respectively. GP is processed and modified by cellular proteases to produce two transmembrane glycoproteins: glycoprotein N (Gn) and glycoprotein C (Gc). The Gc glycoprotein of SFTSV is categorized as a class II fusion protein and facilitates membrane fusion during viral entry, whereas the Gn glycoprotein functions as a surface spike that mediates attachment to host cell receptors, thereby playing a critical role in the initial stages of infection [4].

Several monoclonal antibodies against Gn have been reported to be neutralizing antibodies that inhibit SFTSV infection in vitro [5, 6]. MAb4-5 is a neutralizing antibody that targets SFTSV Gn, which was generated from peripheral blood mononuclear cells (PBMCs) of patients recovered from SFTS in China using a phage display method [5]. S2A5 is a neutralizing antibody that targets SFTSV, which was prepared from BALB/c mice immunized with purified Gn head protein or infectious recombinant vesicular stomatitis virus bearing Gn/Gc (rVSV-eGFP-SFTSV) [6]. The B cells that bind to SFTSV Gn were isolated from the spleens of the immunized mice, and the regions encoding the heavy and light chains of IgG were cloned into a human IgG1 expression vector. To date, these antibodies have not presented sufficient prophylactic or therapeutic efficacy in mouse models of SFTSV infection [6, 7]. Regarding antiviral compounds against SFTS, favipiravir has been approved in Japan in 2024 [8]; however, the therapeutic effects are limited.

Individuals who spend a lot of time outdoors, such as in agriculture and forestry, and who have frequent contact with animals, such as workers in the poultry industry or veterinarians, are at high risk of SFTSV infection [9, 10]. Although a study in the United States showed that SFTSV Gn-specific memory B cells can be detected 174 days after the onset of infection [11], the duration of the study was limited, and the possibility that humoral immunity may persist beyond this time has not yet been fully explored. Determining the duration of humoral immunity against SFTSV and understanding how memory B cells are maintained will provide important insights into predicting the long-term efficacy of convalescent plasma therapy and vaccines.

In this study, blood samples were collected from 16 patients who recovered from SFTS, and PBMCs and plasma were isolated. Using these samples, the status of sustainable humoral immunity was examined.

## Methods

### Cells

HEK293T (CRL-11268; American Type Culture Collection [ATCC]) cells and Vero76 cells (CRL-1587; ATCC) were maintained in Dulbecco’s modified Eagle’s medium (DMEM) supplemented with 10% fetal bovine serum (FBS) and 1% penicillin/streptomycin (PS) solution (NACALAI TESQUE).

### Collection of PBMCs and plasma samples

Patients who were diagnosed with SFTS using the RT-PCR method published by the National Institute of Infectious Diseases, Japan (NIID) [12] and recovered from SFTS after 2015 were recruited from two hospitals in Nagasaki, Japan; 20 mL of blood was collected from 16 patients. For control specimens, blood samples were collected from five healthy adults with no history of SFTS. PBMC and plasma samples were isolated in SepMate tubes (STEMCELL Technologies) after whole blood centrifugation at 1200×*g* for 10 min at room temperature. Plasma samples were stored at − 80 °C until assayed. PBMCs were cryopreserved using Cellbanker1 (Takara) and NALGENE® Mr. Frosty freezing containers (Thermo Fisher Scientific) at − 80 °C and transferred to − 150 °C 24 h later until analysis.

### Plasmid generation

The open reading frame (ORF) encoding GP was amplified by reverse transcription PCR (RT-PCR) from SFTSV (YG-1) viral RNA and inserted into pCAGGS (pCAGGS-SFTSV Gn/Gc (YG1)) using an In-Fusion HD Cloning kit (Takara).

### ELISA

Purified SFTSV Gn protein (Acrobiosystems, GNN-S52H4) was prepared at 1 μg/mL and 100 μL each was added to Nunc Immuno Plate F96 Maxisorp (Thermo Fisher Scientific), incubated overnight at 4 °C, blocked with blocking buffer (PBS-T + 5% goat serum) for 1 h, and washed three times with 300 μL PBS-T. Plasma from SFTS-recovered patients was then serially diluted threefold from 1:50 to 1:36,450, and 100 μL was added to the plate for 1 h at room temperature. The plates were then washed three times with 300 μL PBS-T and diluted with peroxidase AffiniPure® goat anti-human IgG (H + L) (Jackson ImmunoResearch Laboratories, Inc.) in dilution buffer (PBS-T + 1% goat serum) for 30 min. After washing, the TMB substrate (3,3,5,5-tetramethylbenzidine; Thermo Fisher Scientific) was added to the wells, and the plates were incubated for 10 min. The reaction was stopped with 50 μL of 1 M H₂SO₄. The absorbance of each well was measured at 450 and 620 nm to minimize optical interference using a microplate spectrophotometer (Molecular Devices SpectraMax® iD5). Each sample was analyzed in triplicate wells using an ELISA assay to ensure reproducibility and accuracy. The mean optical density (OD) values were calculated, and the standard deviation was used to evaluate intra-assay variability. The assays were carried out in a single experimental run without repeating the entire procedure on the same samples; however, technical replication within the run was applied to ensure measurement reliability.

### Biolayer interferometry (BLI)

The BLI assay was performed using the Octet R2 instrument (Sartorius). Before use, the anti-penta-His (HIS1K) biosensor was pre-hydrated in PBS for 10 min. Flat-bottom 96-well microplates (Greiner, 655201) were filled with 200 μL of patient’s plasma diluted with PBS per well. Assay plates were prepared as follows: column 1 (PBS), column 2 (SFTSV Gn suspended in PBS), column 3 (PBS), and column 4 (plasma diluted 2 × with PBS). SFTSV Gn was loaded onto the HIS-1 K sensor chip for 300 s (s), followed by patient plasma diluted 2× in PBS for 300 s, which resulted in a wavelength shift signal. The Octet Data Analysis software was used for data analysis.

### Construction of VSVΔG-SFTSV Gn/Gc

HEK293T cells were transfected with pCAGGS-SFTSVGn/Gc (YG1) using TransIT LT1 transfection reagent (Mirus). One day after transfection, transfected cells were infected with VSVΔG-VSVG, a recombinant VSV where the VSV-G gene was substituted for a firefly luciferase reporter gene, for 1 h. Twenty-four hours after inoculation, the supernatant was collected, centrifuged at 3000 rpm for 15 min, and stored in a − 80 °C freezer.

### Neutralization assay

Vero76 cells were seeded in 96-well plates and incubated for 24 h. Plasma samples from SFTS convalescent patients and uninfected individuals were thawed and incubated at 56 °C for 30 min to inactivate complement present in the plasma. Serially fourfold diluted plasma was incubated with VSVΔG-SFTSV Gn/Gc for 1 h and then transferred to Vero76 cells. After 24 h, the cells were lysed, and luciferase activity was measured using the Steady-Glo Luciferase Assay System (Promega) and a SpectraMax iD5 microplate reader (Molecular Devices). To confirm the specificity of neutralization, a control assay was performed using VSVΔG-VSVG, and the assay was conducted under the same conditions as for the primary neutralization assay. Each sample was tested in triplicate within a single neutralization assay run to ensure reproducibility. Mean values and standard deviations were used to assess intra-assay variability.

### Flow cytometry

Staining and gating strategies were based on previously published protocols [13], with adjustments for fluorophore combinations and antibody clones. First, PBMCs were incubated with Zombie NIR (BioLegend, 423105) at a 1:1000 dilution at 4 °C for 15 min in fluorescence-activated cell sorting (FACS) buffer (1 × PBS, 2% FCS, and 1 mM EDTA). Next, the following anti-human antibodies were incubated with PBMCs, at 1:100 dilutions: anti-CD20-PECy7 (BD Biosciences, 335793), anti-CD3-APC-eFluro 780 (Invitrogen, 47-0037-41), anti-CD8-APC-eFluor 780 (Invitrogen, 47-0086-42), anti-CD16-APC-eFluor 780 (Invitrogen, 47-0168-41), anti-CD14-APC-eFluor 780 (Invitrogen, 47-0149-42), anti-CD19-BV605 (BD Biosciences, 562654), and anti-CD27-BV711 (BD Biosciences, 564893). PBMCs were also incubated with purified SFTSV Gn conjugated with Alexa Fluor 647 (Thermo Fisher Scientific) at a concentration of 0.372 μg/mL. Flow cytometric analysis was performed using an Attune NxT Flow Cytometer (Thermo Fisher Scientific), and the data were analyzed using FlowJo software. We gated cells that were negative for CD3, CD8, CD14, CD16, and Zombie NIR and positive for CD19, CD27, and SFTSV Gn conjugated with Alexa Fluor 647. PBMCs from four healthy donors with no history of SFTS were used as negative control to ensure reliable and specific measurements. The values observed in the HC group were 0%, 0%, 0%, and 0.021%. From these data, the mean and standard deviation (SD) were calculated (mean: 0.00525%, SD: 0.01047%). The cutoff value was defined as the mean plus two SD (mean + 2SD), which is a commonly used statistical approach to account for background variability and minimize false positives. Therefore, the cutoff value was defined as 0.026%. Accordingly, any sample with a value equal to or above 0.026% was considered positive.

### Statistical analysis

Data were analyzed using GraphPad Prism (version 10.4.2). Continuous variables that were not normally distributed were expressed as medians with interquartile ranges (IQRs), and the Mann–Whitney U test was employed for a nonparametric comparison of the two groups. Spearman’s rank correlation coefficient was used to assess the correlation between two groups. This nonparametric method was chosen because of its robustness in detecting monotonic relationships, including those that are not necessarily linear. FlowJo was utilized for flow cytometry data analysis, and R was used to construct heat maps of the viral neutralization test results.

## Results

### Detection of anti-SFTSV Gn antibodies in the plasma of patients who recovered from SFTS

**Fig. 1:**
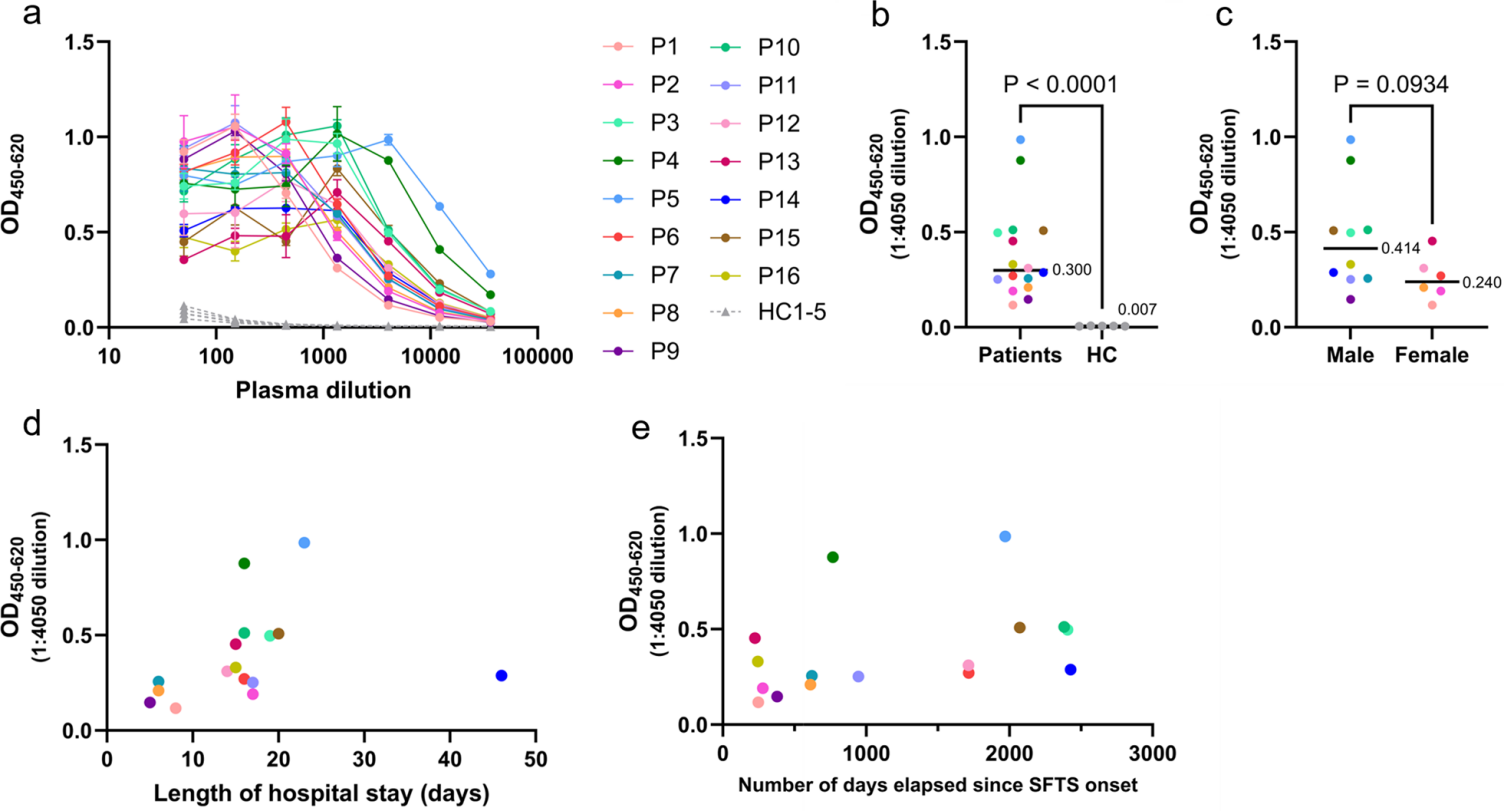
Detection of anti-SFTSV Gn antibodies in the plasma samples from patients who recovered from SFTS by ELISA. **a** OD values were measured by ELISA in plasma samples collected from individual SFTS patients and healthy donors. **b** A comparative analysis of OD values between SFTS patients and healthy donors. Statistical significance was determined using the Mann–Whitney U test, *P* < 0.0001. **c** A comparative analysis of OD values between males and females. **d** Correlation between OD values and length of hospital stay. Statistical analysis was performed using Spearman’s rank correlation coefficient (*ρ* = 0.525, *P* = 0.039). **e** Correlation between OD values and number of days elapsed since onset of SFTS

First, to determine the duration of anti-SFTSV Gn IgG maintenance in individuals who recovered from SFTS, anti-SFTSV Gn IgG in their plasma was detected by ELISA. Data of the patients who recovered from SFTS are summarized in Table 1. For plasma dilutions lower than 1:1,350, several samples showed saturated OD values (Fig. 1a). Therefore, the samples were prepared at a 1:4,050 plasma dilution (Fig. 1b and Table 1). The OD values were not normally distributed, as assessed by the Shapiro–Wilk test (*P* = 0.011). The median OD for the healthy control (HC) group was 0.00676 (IQR [0.00586–0.00811]) (Supplementary Table 1), while the median OD for the patients who recovered from SFTS was 0.288 (IQR [0.209–0.508]). The values for the recovered patient group were higher than those for the HC group in all cases, with statistically significant differences between the groups (*P* < 0.001).

**Table 1:** Clinical and immunological profiles of patients who recovered from SFTS and their samples analyzed in this study

Case no.	Sex	Age at time of blood collection (years)	Age at hospitalization (years)	Days elapsed since onset of SFTS	Length of hospital stay (days)	OD value (1:4050 dilution)	BLI signal at 300 s (nm)	IC₅₀ (dilution)	SFTSV Gn-specific memory B cells (%)
P1	F	69	68	248	8	0.117	0.34	54.6	0.151
P2	F	51	51	279	17	0.191	0.487	133	0
P3	M	75	68	2406	19	0.497	1.432	322	0.198
P4	M	67	65	767	16	0.877	2.946	751	0.107
P5	M	78	72	1970	23	0.985	3.239	1320	0.069
P6	F	77	72	1715	16	0.27	1.14	143	0
P7	M	74	72	620	6	0.256	1.056	224	0.086
P8	F	70	69	611	6	0.209	0.466	55.6	0.052
P9	M	76	75	379	5	0.147	0.457	35.3	0
P10	M	72	65	2383	16	0.512	2.323	104	0.034
P11	M	79	77	946	17	0.252	0.869	212	0.103
P12	F	80	75	1714	14	0.311	1.779	137	0.034
P13	F	78	78	224	15	0.453	2.497	397	0.162
P14	M	57	50	2428	46	0.288	1.507	327	0.079
P15	M	77	71	2073	20	0.508	2.214	256	0
P16	M	64	64	244	15	0.331	0.585	83.2	0.179
Median	M/F = 10/6	74.5 (68.5–77.3)	70.0 (65.0–72.8)	856.5 (354.0–1995.8)	16.0 (12.5–17.5)	0.300 (0.240–0.500)	1.29 (0.560–2.24)	178 (99–323)	0.074 (0.030–0.120)

Furthermore, no statistically significant differences were found between males and females when comparing OD values at the 1:4050 dilution (*P* = 0.0934) (Fig. 1c). However, this result does not rule out the possibility of differences, as the sample size was limited and may not have been sufficiently large to detect slight differences. In the present analysis, the sexes were combined without distinction, as the sex-based differences were not considered statistically significant. Additionally, no correlation was found between the age at blood collection and OD values (*ρ* = 0.205, *P* = 0.447). As it was difficult to use a specific continuous scale to assess illness severity at SFTS onset, the length of hospital stay for each patient was used as a proxy for an indirect method. Spearman’s correlation coefficient showed a positive correlation (*ρ* = 0.525, *P* = 0.039) between the length of hospital stay of each patient and the OD values (Fig. 1d). Lastly, we compared the number of days elapsed since the onset of SFTS, based on ELISA OD values, and found no correlation (*ρ* = 0.479, *P* = 0.062) (Fig. 1e). IgG against SFTSV Gn was detected even in the sample (P14) that was collected 6 years since disease onset, which presented an OD value of 0.288 (Fig. 1e and Table 1).

**Fig. 2:**
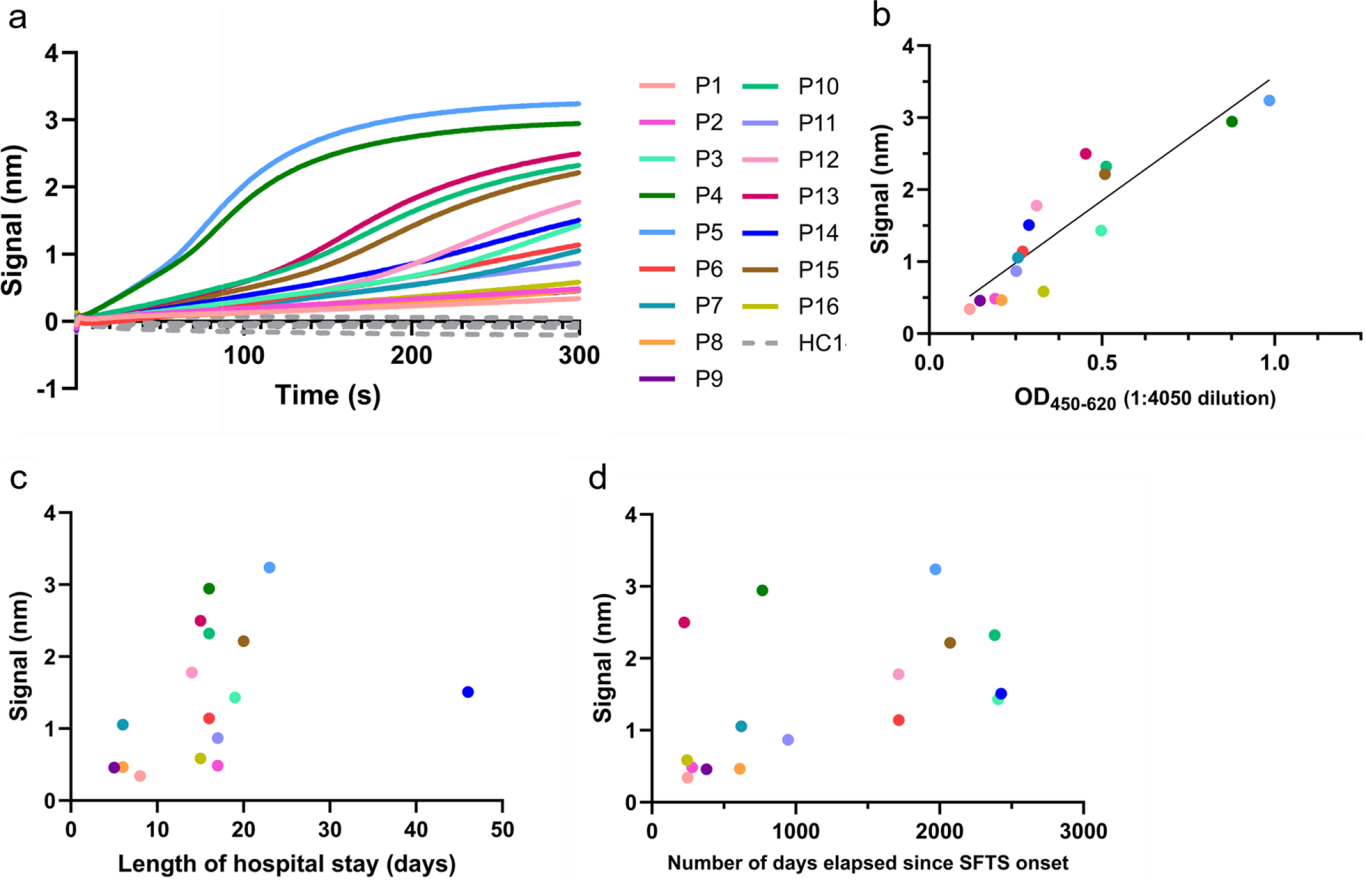
Detection of anti-SFTS Gn antibodies in the plasma samples from patients who recovered from SFTS by biolayer interferometry (BLI). **a** BLI signals were measured in plasma samples from SFTS patients and healthy donors. **b** Correlation between BLI signals and ELISA OD values. Statistical analysis was performed using Spearman’s rank correlation coefficient (*ρ* = 0.813, *P* < 0.0001). **c** Correlation between BLI signals and length of hospital stay. **d** Correlation between BLI signals and number of days elapsed since onset of SFTS

To confirm the ELISA results using another method, a BLI assay was performed to provide a comprehensive assessment of the total amount and binding kinetics of antibodies to SFTSV Gn in the plasma of patients who recovered from SFTS. As the plasma contained polyclonal antibodies and various proteins that could cause nonspecific binding to the sensor, five plasma samples from uninfected individuals were analyzed in the same manner. As with the ELISA results, the patient group exhibited a significantly higher BLI signal than the healthy control group (*P* < 0.001) (Supplementary Table 1). Notably, no signals were detected in the samples from uninfected individuals (Fig. 2a). Under these conditions, all plasma samples from the recovered patients showed binding to SFTSV Gn (Fig. 2a). Furthermore, there was a positive relationship between BLI signals and ELISA OD values (*ρ* = 0.813, *P* < 0.0001) (Fig. 2b). No differences in BLI signals were observed between the sexes (1.470 [IQR 0.798–2.479] vs 0.813 [IQR 0.435–1.959], *P* = 0.313). Similarly, there was no correlation between the number of days elapsed since onset of SFTS and the signals in BLI (*ρ* = 0.474, *P* = 0.066), while a positive correlation was found between the BLI signals and the length of hospital days (*ρ* = 0.525, *P* = 0.039) (Fig. 2c, d). In addition, patient 14, who recovered from SFTS more than 6 years ago, showed a notable anti-SFTSV Gn IgG signal, as observed by ELISA.

### Detection of neutralizing antibodies in the plasma from patients who recovered from SFTS

**Fig. 3:**
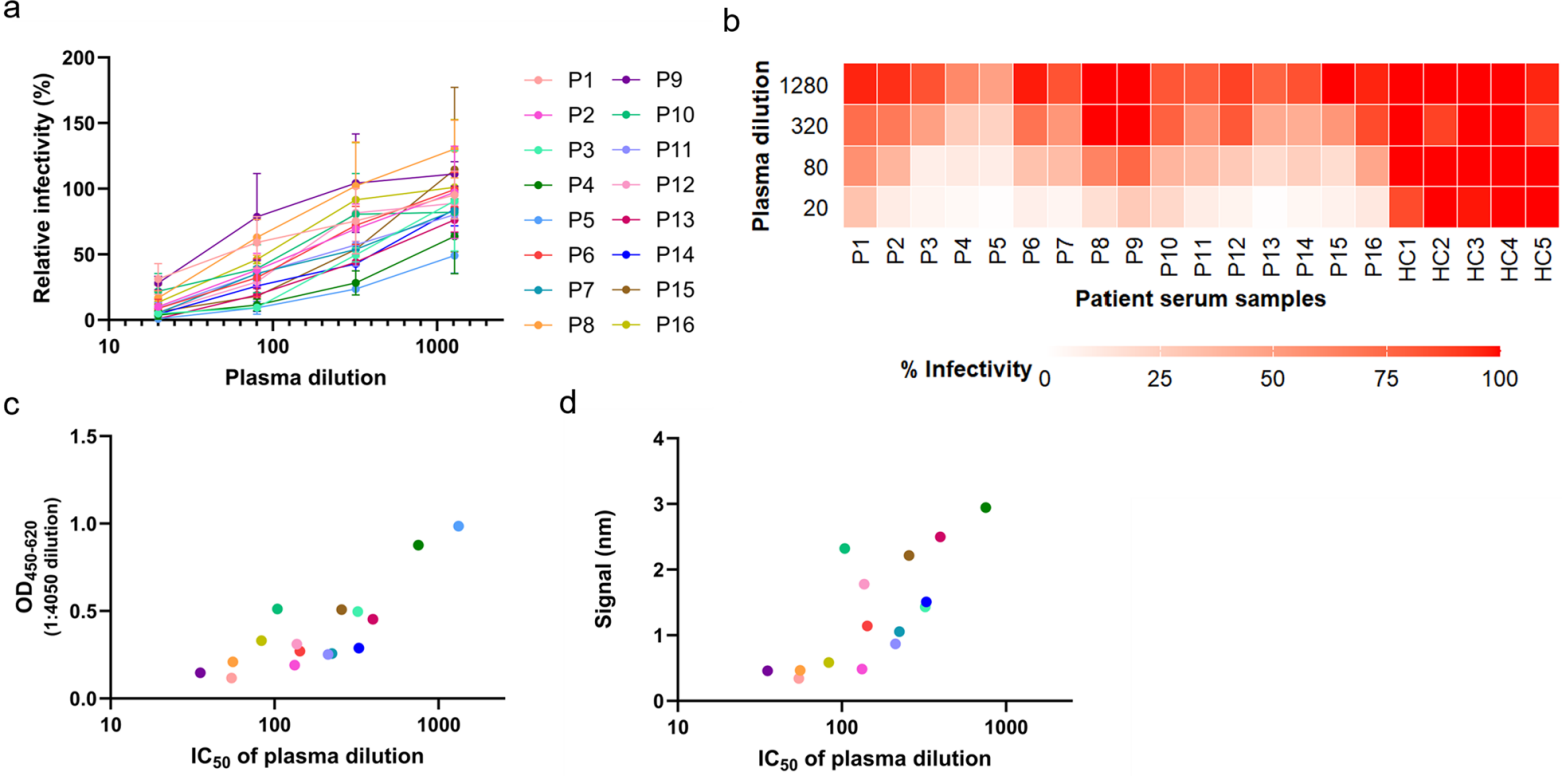
Neutralizing activity against SFTSV of plasma samples. **a** Neutralizing activity of plasma samples against SFTSV was measured. The infection rate of VSVΔG-SFTSV Gn/Gc in the absence of the plasma was set as 100%. Neutralizing activity was determined by the reduction of infectivity at a 1:20–1:1,280 dilution of the plasma. Lower infectivity indicates higher neutralizing antibody activity. **b** The heatmap shows the percentage of infectivity at different plasma dilutions for each patient with SFTS and healthy control. **c** Correlation between ELISA OD values and IC_50_ values in plasma of patients with SFTS. Statistical analysis was performed using Spearman’s rank correlation coefficient (*ρ* = 0.700, *P* = 0.003). **d** Correlation between BLI signals and IC_50_ values in plasma of patients with SFTS. Statistical analysis was performed using Spearman’s rank correlation coefficient (*ρ* = 0.818, *P* < 0.0001)

Both ELISA and BLI assays showed that detectable amounts of anti-SFTSV Gn IgG were present in the plasma of individuals more than 6 years after recovery from SFTS. Next, we examined whether the antibodies detected in the plasma had neutralizing activity against SFTSV infection, and the neutralization assay was performed using a pseudotype VSV bearing SFTSV Gn/Gc (SFTSVpv). As shown in Fig. 3a and b, all plasma samples from patients who recovered from SFTS infection, but not plasma samples from uninfected individuals, exhibited neutralization activities against SFTSVpv. On the other hand, infection by pseudotyped VSV bearing the VSV G glycoprotein was not inhibited by any of the plasma samples from either patients or healthy controls (Supplementary Table 1). These results indicated that all plasma samples from patients who recovered from SFTS infection specifically neutralized SFTSVpv. The relative infection rates of SFTSVpv in the presence of each dilution of plasma from patients who recovered from SFTS infection and healthy controls were also plotted as heat maps (Fig. 3b). The plasma dilutions that showed 50% infectivity were calculated as IC₅₀ (50% inhibitory concentration) values. As shown in Fig. 3c, a positive correlation between the ELISA OD values at 4050-fold dilution and IC_50_ was indicated (*ρ* = 0.700, *P* = 0.003). Similar trends were also observed between BLI signals and IC_50_ (*ρ* = 0.818, *P* < 0.0001) (Fig. 3d). These results altogether suggest that adequate production of neutralizing antibodies is maintained for a long period of time after SFTS recovery and that ELISA and BLI assays using plasma may be suitable for assessing neutralizing antibody levels in patients who have recovered from SFTS.

### Detection of SFTSV Gn-specific memory B cells

As sufficient levels of neutralizing antibodies against SFTSV were detected in patients who recovered from SFTS, we hypothesized that memory B cells, which provide a rapid immune response upon reinfection, would be maintained for an extended period.

**Fig. 4:**
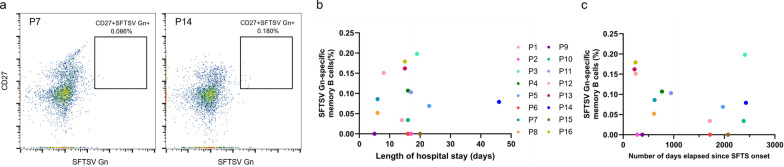
Detection and correlation analysis of SFTSV Gn-specific memory B cells. **a** Frequency of SFTSV Gn-specific memory B cells within CD19^+^ cells in patients with SFTS was determined by flow cytometry analysis. **b** Correlation between memory B cell frequency and length of hospital stay. Statistical analysis was performed using Spearman’s rank correlation coefficient (*ρ* = − 0.002, *P* = 0.995). **c** Correlation between memory B cell frequency and number of days elapsed since onset of SFTS. Statistical analysis was performed using Spearman’s rank correlation coefficient (*ρ* = − 0.212, *P* = 0.427)

To detect SFTSV Gn-specific memory B cells in PBMCs, a flow cytometric analysis was performed and CD3^−^CD8^−^CD14^−^CD16^−^Zombie NIR^−^CD19^+^CD27^+^SFTSV Gn^+^ cells were gated (Fig. 4a). HC samples served as negative controls, with a positivity cutoff set at 0.026% (mean + 2 SD). For each patient who recovered from SFTS, the percentage of CD27^+^SFTSV Gn^+^ cells in CD19^+^ cells was calculated as the percentage of memory B cells specific for SFTSV Gn. The median percentage of SFTSV Gn-specific memory B cells within total B cells was 0.074% (IQR [0.009–0.140]), and memory B cells in SFTSV Gn were detected in 12 of the 16 patients who recovered from SFTS (Table 1). There was no significant correlation between the frequency of SFTSV Gn-specific memory B cells and ELISA OD values (*ρ* = 0.193, *P* = 0.471) or BLI signals at 300 s (*ρ* = 0.071, *P* = 0.793). These results suggest that even though memory B cells specific for SFTSV Gn in the peripheral blood of patients who recovered from SFTS infection were undetectable in flow cytometric analysis, antibodies to SFTSV Gn continued to be produced by plasma cells. There was also no association between the length of hospital stay nor the days elapsed since the SFTS onset (*ρ* = − 0.002, *P* = 0.995) and the possession of memory B cells (*ρ* = − 0.212, *P* = 0.427) (Fig. 4b, c). Importantly, memory B cells specific for SFTSV Gn were detected in patients 6 years after onset, thus suggesting that the cells persist for a long duration.

## Discussion

In this study, neutralizing antibodies against SFTSV were detected in 16 of 16 recovered patients, and previous studies have shown that all 25 recovered patients retained neutralizing antibodies for at least 4 years [14]. Furthermore, neutralizing antibodies have been found to reach 100% positivity at 6 months post-illness, remain stable for 2 years, and gradually decline to 89.5% at 3 years, 78.3% at 5 years, and 66.7% at 10 years [15]. In our study, from the three samples that were collected from individuals more than 6 years since disease onset, sufficient amounts of neutralizing antibodies to inhibit SFTSV infection were detected (Fig. 3a and b), which suggests that SFTSV infection induces durable humoral immunity. Furthermore, the long-term presence of neutralizing antibodies may reduce the risk of reinfection in high-risk populations such as livestock and forestry workers, veterinarians, and pet owners. While long-term neutralizing antibody responses may aid in preventing reinfection, antibody-dependent enhancement (ADE) remains a theoretical concern in viral infections, including SFTSV. ADE occurs when sub-neutralizing or non-neutralizing antibodies facilitate Fc receptor-mediated viral entry into host cells, potentially worsening disease severity [16, 17]. However, no cases of ADE have been reported for SFTSV or closely related viruses in the bunyavirus family to date. Furthermore, the neutralization assays employed utilized Vero cells, which are deficient in Fcγ receptors. Consequently, the assessment of ADE was not feasible within the confines of this experimental system. Future studies employing FcγR-expressing cell lines or in vivo animal models are warranted to assess the potential for ADE in SFTSV infection.

Detailed epidemiological data regarding the prevalence of SFTS are limited. Between 2011 and 2021, 18,902 confirmed cases and 966 deaths were reported in China, which corresponds to a cumulative incidence of 0.13 cases per 100,000 persons [18]. Reinfection appears to be rare; however, a case involving a 42-year-old woman in Henan Province, China, infected with a different SFTSV strain, was reported in 2021 [19]. Furthermore, in a previous study, two of 25 patients who recovered showed higher neutralizing antibody titers at 4 years post-infection than at 1 year [14]. In our study, ELISA and BLI revealed that antibody titers did not show a declining trend over time since the onset of illness (Fig. 1c). Although none of these patients have had multiple episodes of SFTS, these findings suggest that some patients may experience reinfection or antigen re-exposure, thus leading to increased production or maintenance of neutralizing antibodies against SFTSV. However, sufficient evidence for this remains to be fully established.

A previous study indicated that patients with severe SFTS tended to exhibit higher IgG levels than moderately symptomatic patients and showed a slower decline in antibody titers over time [20]. Furthermore, it has been reported that disease severity positively correlates with viral load at the time of illness onset [21]. In this study, we also observed a positive correlation between the length of hospitalization and neutralizing antibody levels, which suggests that patients with more severe disease had more robust antibody responses. These findings support the hypothesis that intense immune stimulation during severe infections may lead to stronger and more durable humoral immunity in convalescent patients.

Convalescent plasma therapy has been considered for other viral diseases, such as Coronavirus disease 2019 (COVID-19) and Ebola virus disease [22, 23]. In previous studies that used an IFNAR knockout (α/β interferon receptor-deficient) mouse model highly susceptible to SFTSV, post-exposure prophylaxis with human antiserum provided complete protection against lethal infection [24]. In South Korea, convalescent plasma therapy administered to patients with SFTS has been reported to improve survival by reducing viral RNA levels in the plasma over time [25]. The long-term persistence of neutralizing antibodies in patients who recover from SFTS suggests that blood donation from a broad range of recovered individuals across generations may be feasible, thereby expanding the potential for therapeutic plasma or serum use.

In this study, we successfully used BLI to detect antibodies specific for SFTSV Gn in patients with recovered SFTS, and BLI has several advantages over ELISA. First, sample preparation was easily completed in less than 30 min, and no manual intervention was required until completion of the analysis. Second, molecular interactions can be observed in real time. Notably, BLI has been shown to be capable of quantitative antibody detection in patients infected with severe acute respiratory syndrome coronavirus 2 (SARS-CoV-2) [26]; therefore, this detection system would be versatile regardless of the virus species.

Memory B cells play a crucial role in enhancing the immune response upon re-exposure to pathogens. Upon encountering antigens, these cells rapidly differentiate into antibody-secreting cells or re-enter germinal centers for further diversification and affinity maturation [27]. Consequently, the ability to elicit virus-specific memory B cells is pivotal for the formulation of effective vaccines. In the context of COVID-19 and Ebola virus disease, neutralizing monoclonal antibodies have been developed as antivirals [28, 29]. While the development of MAb4-5 for SFTS did not contribute to the treatment of SFTS in experimental mouse models [30], Ab10, a humanized monoclonal antibody targeting SFTSV Gn, showed 80% protection when administered five days post-infection in a mouse model [30]. However, Ab10 requires a high dose of 30 mg/kg to protect against SFTSV infection in mice and was validated only in Gangwon/Korea/2012 strain [30]. In other studies, the RNA polymerase inhibitor favipiravir showed efficacy in preventing and treating SFTSV infection in animal models [31, 32] but failed to adequately reduce mortality in a multicenter, nonrandomized, uncontrolled, single-arm study of patients with resolved SFTS [8]. Currently, there are no effective treatments for SFTS. In this study, memory B cells specific for SFTSV Gn were detected in PBMCs from 75% (12/16) of patients who recovered from SFTS and persisted for up to 6 years and 7 months post-infection (Fig. 4c). Therefore, the generation of recombinant neutralizing monoclonal antibodies from memory B cells may contribute to the development of novel anti-SFTS therapies.

Memory B cells specific for SFTSV Gn were not detected in 4 of 16 patients; however, neutralizing antibodies remained present in their plasma. This observation suggests that the maintenance of antibody responses may not be solely dependent on circulating memory B cells. A previous study using non-human primates showed that the depletion of memory B cells had no impact on bone marrow plasma cell numbers and serum antibody titers for prolonged periods and indicated that long-lived plasma cells (LLPCs) can maintain humoral memory independently of memory B cells [33, 34]. Therefore, LLPCs may sustain antibody production in the bone marrow, spleen, or lymph nodes. Further investigation is warranted to characterize the anatomical niches and survival mechanisms of these LLPCs. Such insights would enhance our understanding of long-term immune protection against SFTSV and inform the development of more effective and durable vaccine strategies.

This study has several limitations. First, the patients in the acute phase of illness were not included; therefore, the data on immune responses during this phase are lacking. Second, longitudinal follow-up was not performed, which limited the ability to evaluate temporal changes in immunity. Third, the study population was small, comprising only 16 patients from a single prefecture, which reduced statistical power, particularly for subgroup analyses by sex and age. In addition, the analyses such as ELISA, BLI, and flow cytometry targeting SFTSV Gc protein were not performed, which could have provided complementary insights into humoral and cellular immune responses. Consequently, the generalizability of these findings to broader patient populations is limited. We expect that future studies with larger, more diverse cohorts, longitudinal sampling, and comprehensive immunological profiling—including both Gn and Gc-specific responses—will help to overcome these limitations and further clarify the immunological landscape of SFTSV infection.

## Conclusions

In summary, the present study elucidates the long-term persistence of humoral immune responses against SFTSV in recovered individuals. Quantitative analyses by ELISA and BLI revealed sustained levels of SFTSV Gn-specific antibodies over extended periods. A neutralization assay was used to assess the functional activity of VSVΔG-SFTSV Gn/Gc. This assay demonstrated consistent neutralizing activity across all plasma samples from patients, with the IC_50_ values exhibiting a strong correlation with the ELISA OD value and BLI signal. It is noteworthy that SFTSV Gn-specific memory B cells could be detected by flow cytometry in 12 out of 16 individuals, including those as late as 6.7 years post-infection. Collectively, these findings indicate that SFTSV-specific humoral immunity, encompassing both neutralizing antibodies and memory B cells, is maintained for prolonged durations following recovery. The sustained humoral immunity observed in this study underscores the potential utility of convalescent plasma as a therapeutic modality and provides a scientific foundation for the development of effective vaccines targeting SFTSV.

## Supplementary Information

Supplementary material 1.

## Data Availability

No datasets were generated or analyzed during the current study.

## Abbreviations

BALB/c mice: Bagg's Albino c mice (inbred laboratory mouse strain)
BLI: Biolayer interferometry
ELISA: Enzyme-linked immunosorbent assay
GFP: Green fluorescent protein
Gc: Glycoprotein C (envelope glycoprotein C of SFTSV)
Gn: Glycoprotein N (envelope glycoprotein N of SFTSV)
IC_50_: 50% Inhibitory concentration
IFNAR: Interferon alpha/beta receptor
OD: Optical density
PBMCs: Peripheral blood mononuclear cells
SFTS: Severe fever with thrombocytopenia syndrome
SFTSV: Severe fever with thrombocytopenia syndrome virus
VSV: Vesicular stomatitis virus
LLPCs: Long-lived plasma cells
