# Supplementary material for: Sustained humoral immunity in the patients recovered from severe fever with thrombocytopenia syndrome
Source: Trop Med Health. 2025 Sep 30;53:127. doi: 10.1186/s41182-025-00807-4 (PMC12487157; doi:10.1186/s41182-025-00807-4)
Supplement: Supplementary file 1 — Supplementary material 1. [file 41182_2025_807_MOESM1_ESM.docx]

**Supplementary Table 1. Results of ELISA and BLI assays for the healthy control group.**

| Case no. | Age at time of blood collection (years) | OD value (1:4050 dilution) | BLI signal at 300 s (nm) | SFTSpv  IC₅₀ (dilution) | VSVΔG-G  IC₅₀ (dilution) | SFTSV Gn-specific memory B cells (%) |
| --- | --- | --- | --- | --- | --- | --- |
| HC1 | 30 | 0.00586 | -0.0875 | ND^*^ | ND^*^ | – |
| HC2 | 35 | 0.00811 | 0.0495 | ND^*^ | ND^*^ | 0.000 |
| HC3 | 53 | 0.00586 | -0.0742 | ND^*^ | ND^*^ | 0.000 |
| HC4 | 40 | 0.00699 | -0.0314 | ND^*^ | ND^*^ | 0.000 |
| HC5 | 33 | 0.00676 | -0.2030 | ND^*^ | ND^*^ | 0.021 |
| Median  (IQR) | 35 (33.0–40.0) | 0.00676 (0.00586–0.00699) | -0.0742 (-0.0875–-0.0314) | – | – | 0 (0–0.0105) |

* ND (Not determined): IC₅₀ could not be determined since the compound showed less than 50% inhibition within the tested concentration range.
